# A novel adapted MRI-based scheme for Dejour classification of trochlear dysplasia

**DOI:** 10.1007/s00256-024-04748-7

**Published:** 2024-07-23

**Authors:** Ni Jian-Lüssi, Christian W. A. Pfirrmann, Florian M. Buck, Thomas Frauenfelder, Andrea B. Rosskopf

**Affiliations:** 1 Faculty of Medicine, University of Zurich, Zurich, Switzerland; 2 Medical Radiological Institute (MRI) Zurich, Schulthess Clinic, Lengghalde 2, CH-8008 Zurich, Switzerland; 3 Institute of Diagnostic and Interventional Radiology, University Hospital Zurich, Rämistrasse 100, 8091 Zurich, Switzerland

**Keywords:** Trochlear dysplasia, MRI, Classification, Dejour, Knee

## Abstract

**Purpose:**

To elaborate an optimized scheme for the Dejour classification of trochlear dysplasia based on axial and sagittal MR images and to evaluate its intra- and inter-reader reliability.

**Material and methods:**

Over a period of 20 months patients with a knee MRI and the diagnosis of trochlear dysplasia were retrospectively included. Exclusion criteria were incomplete examination, qualitatively non-diagnostic examination, post trochlear surgery, missing informed consent for research purposes. Three independent evaluations were performed by two radiologists: first using an established description of the Dejour classification (types A–D) and then two evaluations using a new adapted scheme (types A–D). The adapted scheme includes a shallow trochlea, in type A no spur/no cliff, in type B with spur/no cliff, in type C no spur/with cliff, and in type D with spur/with cliff.

**Results:**

One hundred seventy-one knee MRIs (female:65.5%; left side:52.6%) were included with a median age of 34.3 years (range:11.3–79.2). Inter-reader reliability using the established description was fair for the four-type-classification (kappa(*k*) = 0.23; 95%CI:0.11–0.34), fair for differentiation low-grade versus high-grade dysplasia (*k* = 0.28;0.13–0.43), slight for differentiation spur versus no-spur types (*k* = 0.20;0.05–0.34). Inter-reader reliability using the adapted scheme was substantial (*k* = 0.79;0.75–0.83) for the four-type-classification, substantial for differentiation low-grade versus high-grade dysplasia (*k* = 0.80;0.75–0.85), substantial for differentiation spur versus no-spur presence (*k* = 0.76;0.71–0.81). Intra-reader reliability was almost perfect for the adapted scheme (*k*-values: 0.88–0.95; 95%CIs: 0.84–0.98).

**Conclusion:**

The novel adapted scheme for Dejour classification shows an almost perfect intra-reader reliability and a substantially higher inter-reader reliability. It may become a helpful tool in the daily diagnostic work of radiologists.

## Introduction

Trochlear dysplasia refers to an abnormal trochlear morphology characterized by a shallow groove and is known to be a major risk factor for patellofemoral instability. The exact type of trochlear dysplasia is important for surgical treatment planning, e.g., whether a medial patellofemoral ligament reconstruction is performed alone or in combination with a trochleoplasty [1].

Trochlear dysplasia was first described on strictly lateral conventional radiographs by Maldague and Malghem 1985 [2]. In 1987, Henri Dejour defined a classification of trochlear dysplasia based on the level of the crossing sign on lateral knee radiographs [3–5]. In 1998 his son David Dejour expanded this classification to also include the supratrochlear spur and double-contour sign by cross-referencing lateral radiographs with axial slices on computed tomography (CT) imaging [6, 7]. Nowadays, the Dejour classification is most commonly used to categorize and describe trochlear dysplasia: it refers to 4 different types (A, B, C, and D) of trochlear dysplasia [7]. The Dejour classification from 1998 has been criticized for having a poor intra- and inter-observer agreement [8–10]. Moreover, the description of the criteria for analyzing the axial image differ from the original publication and have changed over the years. Currently, there is no clear standard for the application of Dejour classification based on magnetic resonance imaging (MRI), and different authors have used different methods, resulting in poor agreement between conventional radiography and MRI and low reproducibility [11–15]. The adapted Table 1 probably represents the consensus for the Dejour classification on radiographs and axial images today. Experienced musculoskeletal radiologists in our department also reported uncertainties and poor reproducibility when using the Dejour classification for MRI in clinical routine. Therefore, the aim of our study was to simplify and standardize the 1998 Dejour classification for MRI purposes by using axial and sagittal MRI planes to achieve better intra- and inter-rater reliability.

**Table 1:** Trochlear dysplasia, Dejour grading, findings on lateral radiographs and axial images

Original		Lateral radiograph	Axial cross-sectional Image
Simple dysplasia	A	Crossing sign	Shallow trochlea
Simple dysplasia with supratrochlear spur	B	Crossing sign + Supratrochlear spur	Flat or convex trochlea
Complex dysplasia	C	Crossing sign + Double contour	Asymmetry of the trochlear facets lateral facet convex with hypoplastic medial facet
Complex dysplasia with supratrochlear spur	D	Crossing sign + Supratrochlear spur + Double contour	Asymmetry of the trochlear facets vertical slope demonstrating a "cliff" pattern

Adapted from Kazley JM, Banerjee S. Classifications in Brief: The Dejour Classification of Trochlear Dysplasia [9]. The left column shows the description in the original paper by D. Dejour in 1998. The three right columns show the current established criteria for Dejour classification types A–D

In contrast to the cross-sectional images, the specifications for the evaluation of lateral standard radiographs are straight forward uniform and well accepted in various subsequent publications [13, 16, 17]:. Crossing sign: The crossing sign is the radiographic representation of the deepest point of the trochlear groove crossing the anterior border of the femoral condyles. Supratrochlear spur: A supratrochlear spur is a protrusion of the proximal border of the trochlea above the anterior cortex of the femur. Double contour: A double contour is present when the medial facet is hypoplastic or missing. The anterior line of the double contour is the anterior edge of the lateral condyle, the posterior line represents the cortex of the femur, as the medial facet is not present. A double contour caused by the absent medial facet corresponds to the cliff pattern on the axial image

## Materials and methods

This retrospective study was approved by the local ethics committee (BASEC No. 2022-01534). Our institutional PACS system (Carestream Vue PACS, Version 12.2.2.1025, Carestream Health, Inc, Rochester, NY, USA) was searched between December 2020 and August 2022 for patients with a knee MRI examination and the RIS system (MEDAVIS RIS, Version 5.3.7.2, Karlsruhe, Germany) for diagnosis of trochlear dysplasia (by a radiologist not involved in the following evaluations: ABR) in the corresponding report. All initial MRI reports were done by board-certified radiologists with more than 8 years of experience in MSK radiology. The following inclusion criteria were applied: age between 6 and 99 years, knee MRI examination between December 2020 and August 2022, and diagnosis of “trochlear dysplasia” in the conclusion of the MRI report. Exclusion criteria were as follows: incomplete examination, qualitatively non-diagnostic examination, post trochlear surgery, and missing informed consent for research purposes.

## MRI examination

All patients were scanned in 1.5 or 3 Tesla scanners (Sola/Avanto/Aera or Vida/Skyra; Siemens Healthcare, Erlangen Germany) with the institutional routine knee MRI protocol, an example protocol for the Sola scanner can be found in Table 2. The parameters for the protocols of the other scanners were slightly adapted according to scanner characteristics.

**Table 2:** Routine protocol for native 3 T-MRI of the knee in our institution

Sequence	FoV (mm)	Slice thickness (mm)	TE (ms)	TR (ms)
Coronal PDfs	160	3	43	4030
Coronal T1	160	3	13	566
Sagittal PDfs	160	3	37	3960
Sagittal PD	160	3	17	2990
Transverse PDfs	160	2.5	38	5440

*FoV*, field of view; *TE*, echo time; *TR*, repetition time; *ms*, milliseconds; *PDfs*, proton density fat saturated

## Image evaluation

**Fig. 1:**
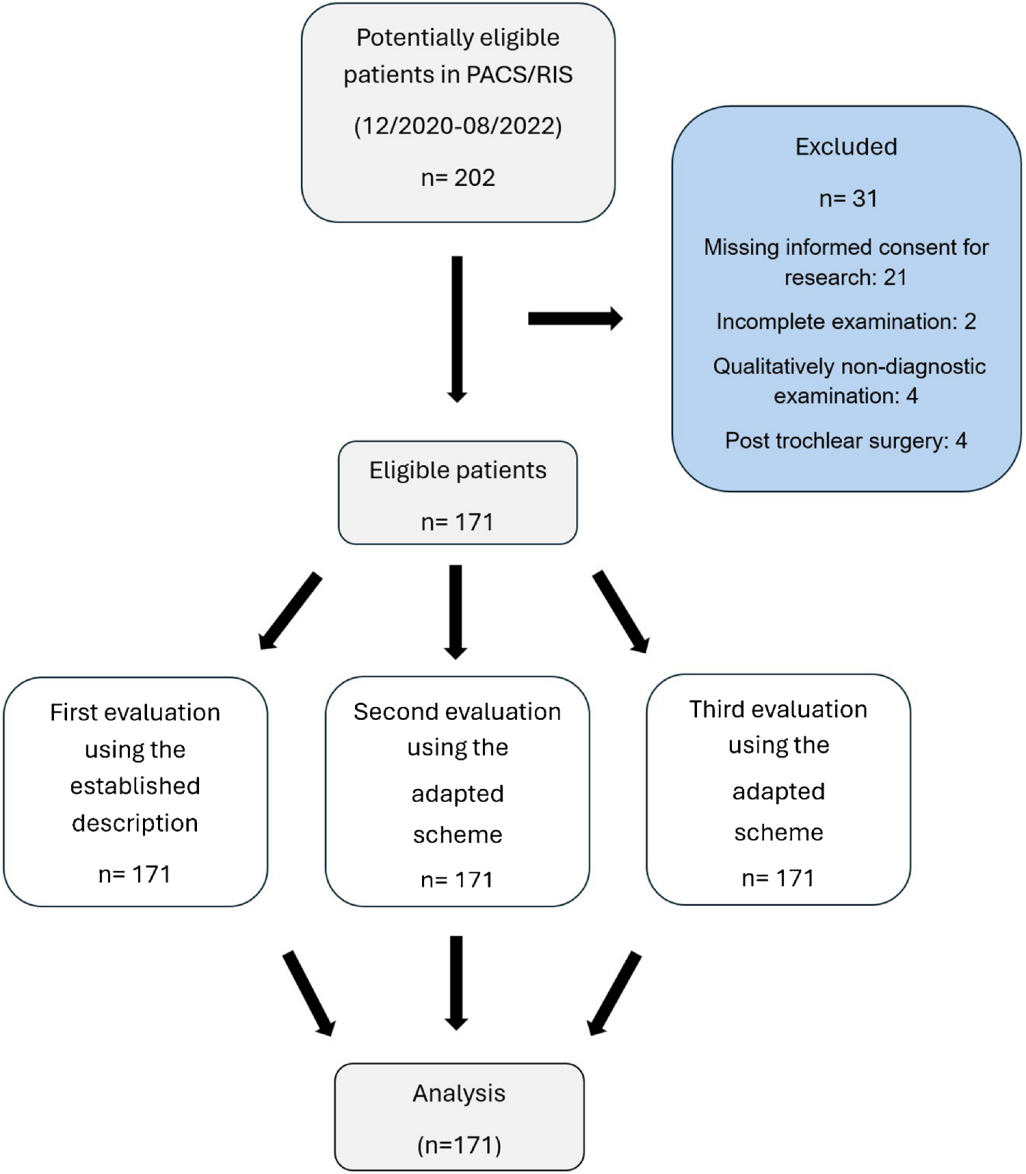
Study flowchart

Two radiologists (NJL (Reader 2) with 5 years of experience in MSK radiology and FMB (Reader 1) with 18 years of experience in MSK radiology) performed three evaluations of the cohort (4 months between each evaluation to exclude memorization of images). The readers were blinded to the original MRI reports. The study flowchart is shown in Fig. 1.

## First evaluation (current description of Dejour classification)

In the first evaluation, the two readers assessed the trochlear dysplasia according to the Dejour classification types A–D using the current established criteria and illustrations in the paper by Kazley and Banerjee (see Table 1 in this study and Fig. 4 in Reference [9] – weblink in References):

- Type A: fairly shallow trochlea
- Type B: flat or convex trochlea
- Type C: asymmetry of trochlear facets with a hypoplastic medial condyle
- Type D: asymmetry of trochlear facets plus vertical joint/cliff pattern

The entire distal femur was analyzed by the two readers (not only a specific slice).

## Second evaluation (adapted scheme)

In the second evaluation (4 months after the first evaluation), the two readers classified the knees again analyzing the entire distal femur on axial and sagittal MRI slices using the following criteria.

### Novel adapted scheme according to the presence of a spur or cliff (see Fig. 2)

**Fig. 2:**
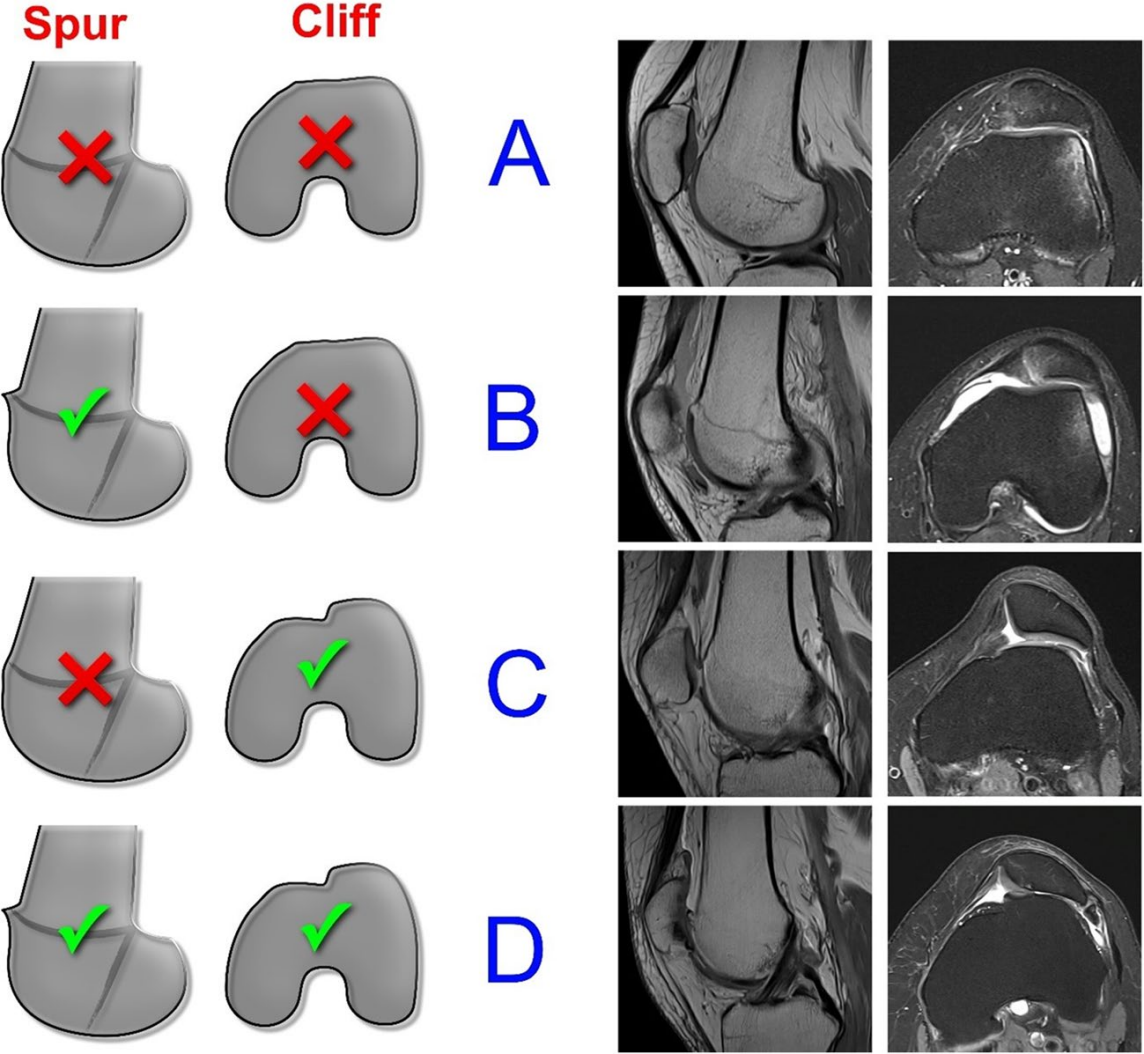
Novel adapted classification scheme. Adapted scheme showing each classification category (A, B, C, or D) with illustrations on the left side and corresponding MRI examples (sag proton-density (pd) weighted and axial proton-density-with-fat-saturation (pdfs) weighted) on the right side. Checkmark means the finding is present. Cross sign (X) means the finding is absent

- Type A: shallow trochlea, spur: no, cliff: no
- Type B: shallow trochlea, spur: yes, cliff: no
- Type C: shallow trochlea, spur: no, cliff: yes
- Type D: shallow trochlea, spur: yes, cliff: yes

### Definition of a spur presence

**Fig. 3:**
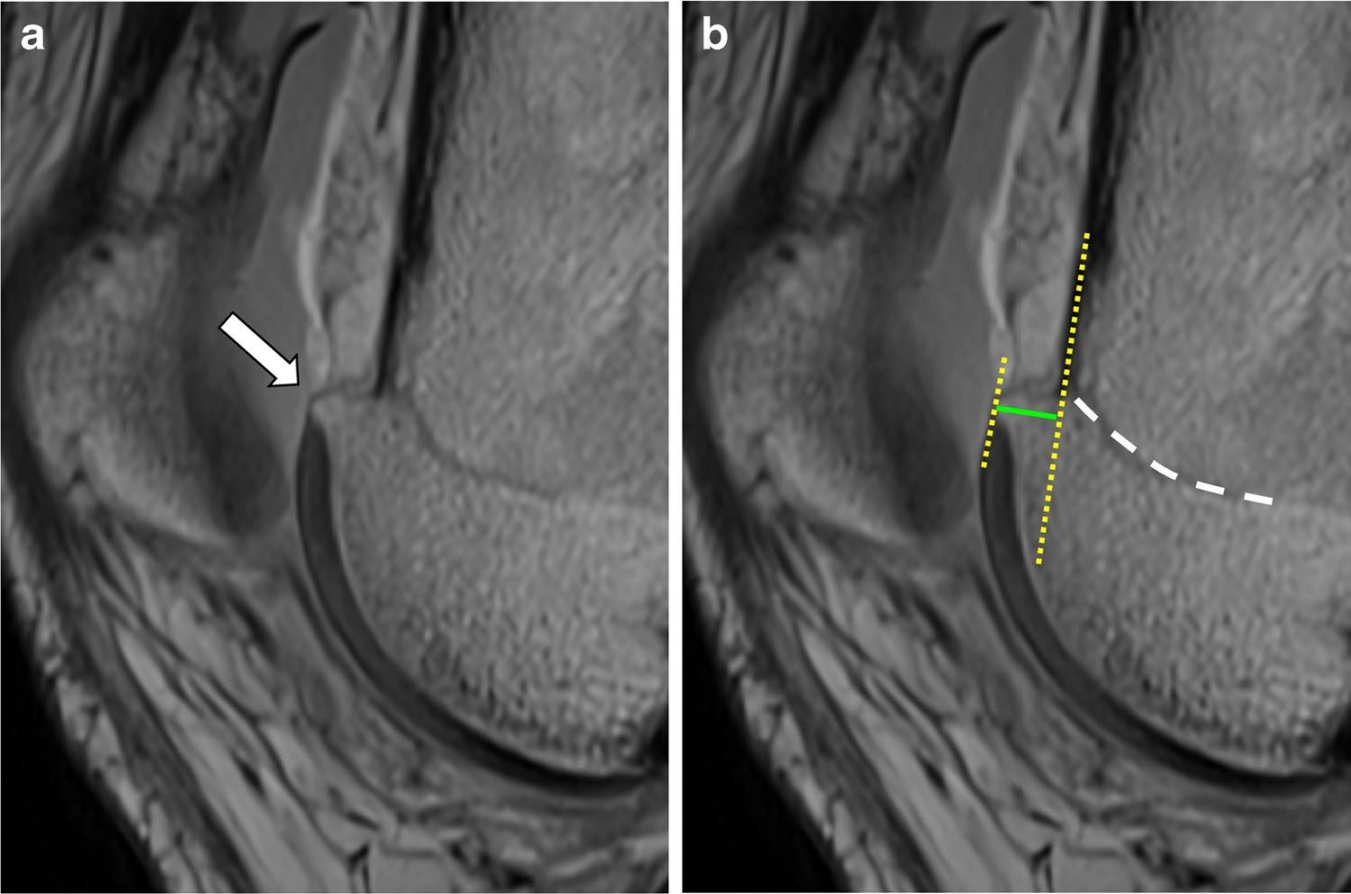
Presence of a spur (arrow) on mid-sagittal proton-density-weighted image in a patient with trochlear dysplasia. Same image (B) shows measurement of spur height by drawing a tangent at the anterior cortical bone of the trochlea and a parallel line at the spur tip (yellow dotted lines). The distance between the lines measures the height of the spur (green line). Note the level of the epiphysis (dotted white line) adjacent to the spur

- Visible spur on mid-sagittal (= within the second and third quarter of the trochlea) at level of the epiphysis: yes/no
- Clear spur (> 2.5 mm)
- Spur size measured in mm: maximal distance from tangent at anterior trochlear cortex (see Fig. 3)

### Definition of a cliff presence

**Fig. 4:**
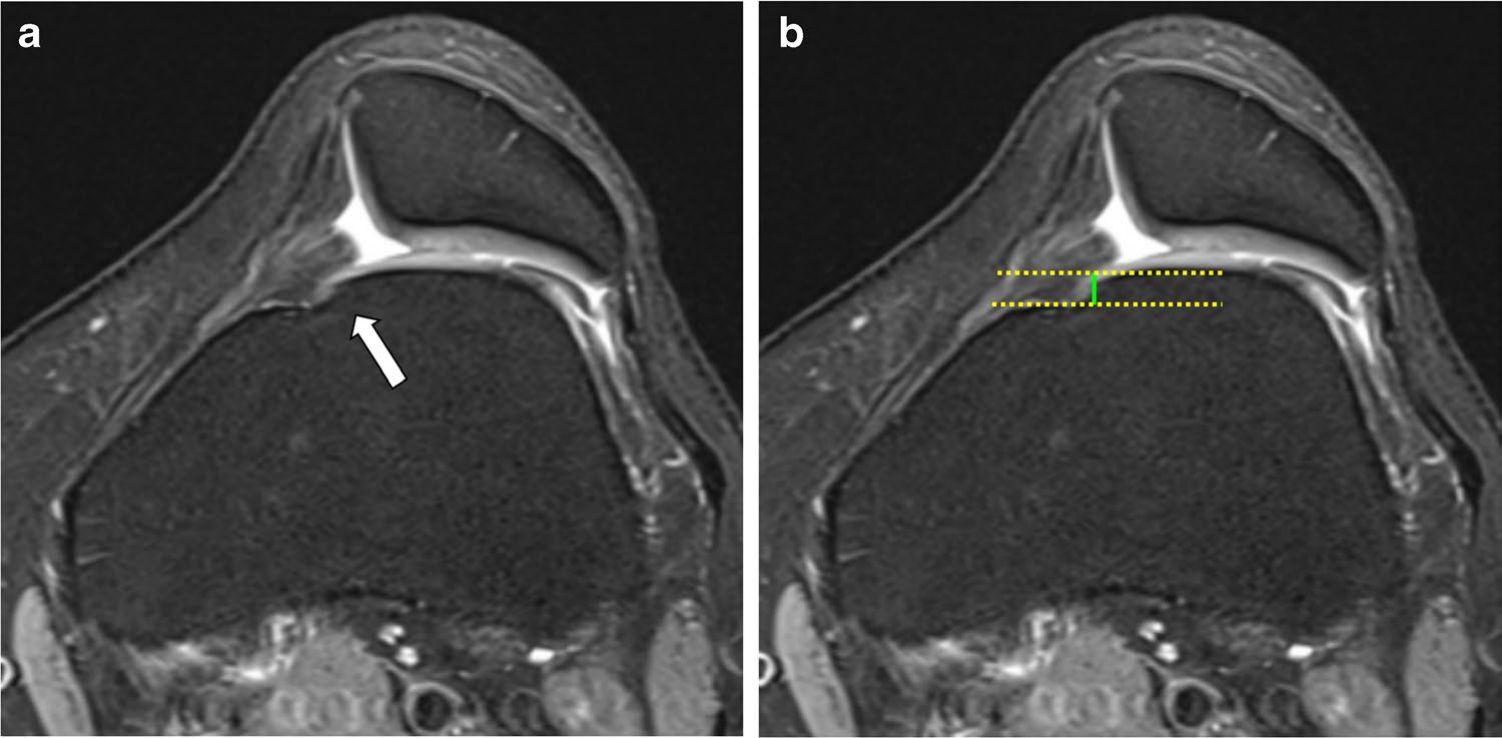
Axial pdfs (**A**) showing small cliff (arrow) in a patient with trochlear dysplasia. Same image (**B**) showing the measurement of cliff by drawing a horizontal line adjusted to the bony contour of the medial trochlea and parallel line to the cortical bone of the central trochlea (yellow dotted lines). The distance between the lines measures the height (in mm) of the cliff (green line)

- Visible cliff (> 3.0 mm) on transverse plane: yes/no
- Step-like transition from lateral facet to anterior cortical bone of the femur
- Cliff height in mm (maximum on transverse images (see Fig. 4))

## Third evaluation (adapted scheme)

Four months after the second evaluation, the two readers assessed the included knees again (using only the adapted classification) in order to calculate intrareader reliability for the adapted classification.

## Statistics

Study data were collected and managed using REDCap® electronic data capture tools (Version 13.5.4; Vanderbilt University, USA) hosted at the Schulthess Clinic, Zurich, Switzerland [18]. Descriptive statistics for basic demographic data and summary of evaluations per reader were created using the “Stats & Charts”-Tool in RedCap®: age (years: mean and range), gender (%), side (left in %), dysplasia type (A, B, C, or D in %) and spur and cliff sizes (mean ± SD). The inferential analysis was conducted with SPSS (Version 28.0. Armonk, NY: IBM Corp) and MATLAB (The MathWorks Inc. (2022b), Natick, Massachusetts). *P*-values below 0.05 were considered statistically significant.

Cohen’s kappa values for inter-reader and intra-reader reliability were interpreted as follows: 0.01–0.20 indicating slight agreement beyond chance, 0.21–0.40 indicating fair agreement beyond chance, 0.41–0.60 indicating moderate agreement beyond chance, 0.61–0.80 indicating substantial agreement beyond chance, 0.81–1.00 indicating almost perfect agreement beyond chance [19, 20]. Inter-reader reliability of continuous measurements (i.e., spur and cliff size) was quantified in terms of the intraclass correlation coefficient (ICC) [21] based on a two-way random effects model assessing the absolute agreement of a single-measure approach and the related standard error of measurement (SEM). ICC values were interpreted as follows: Values less than 0.5 indicate poor reliability, values between 0.5 and 0.75 indicate moderate reliability, values between 0.75 and 0.90 indicate good reliability, and values greater than 0.9 indicate excellent reliability. In the two categories for cases with a positive finding of a bone spur, we assessed potential differences in spur size based on the coincident presence or absence of a bony cliff (i.e., category B vs. D). The analogous procedure was applied to assess for a difference in cliff size—provided it is present—as a function of the presence or absence of a cliff (i.e., category A vs. C). These comparisons were conducted using nonparametric tests (Mann Whitney *U*-tests; two-sided).

## Results

One hundred seventy-one knees of 161 patients (65.5% female) were included as patient cohort with a median age of 34.3 years (range 11.3–79.2 years). In 47.4% of knees the right side and in 52.6% the left side was examined (see Table 3).

**Table 3:** Basic demographics and summary of evaluations

Variable	n (%)			
Knees: *n* (%)	171 (100)			
Age in years	34.3 (11.3–79.2)*			
Female	112 (65.5)			
Left side	90 (52.6)			
Dejour types	Established description (Evaluation 1)		Adapted scheme (Evaluation 2)	
	Reader 1 *n* (%)	Reader 2 *n* (%)	Reader 1 *n* (%)	Reader 2 *n* (%)
A (low grade)	60 (35.1)	68 (39.8)	83 (48.5)	83 (48.5)
B	79 (46.2)	91(53.2)	55 (32.2)	51 (29.8)
C	28 (16.4)	10 (5.8)	12 (7.0)	16 (9.4)
D	4 (2.3)	2 (1.2)	21 (12.3)	21 (12.3)
High grade (B–D)	111 (64.9)	103 (60.2)	88 (51.5)	88 (51.5)
A and C (no spur)	88 (51.5)	78 (45.6)	95 (55.5)	99 (57.9)
B and D (spur)	83 (48.5)	93 (54.4)	76 (45.5)	72 (42.1)
Spur size of types B and D (mm, mean ± SD)	-		3.7 ± 1.5	3.6 ± 1.4
Cliff size of types C and D (mm, mean ± SD)			4.1 ± 1.1	4.3 ± 1.1

^*^Mean (range)

This patient cohort was used for all evaluation rounds (1–3). The readout results are presented using the 4-type trochlear dysplasia classification (types A–D), the 2-type trochlear dysplasia classification of low (type A) and high grade (types B, C, and D), and whether a supratrochlear spur was present or not (types A and C versus types B and D).

High-grade dysplasia was more often rated using the established description (reader 1: 64.9%; reader 2: 60.2%) than with the adapted scheme (both readers: 51.5%). Using the adapted scheme, type A was most often rated both by reader 1 (48.5%) and 2 (48.5%). All other detailed results for Evaluations 1 and 2 can be found in Table 3.

Table 4 shows that classification differences between the readers were much higher in Evaluation 1 (established description) compared to Evaluation 2 (adapted scheme). Therefore, inter-reader reliability was much higher for the adapted scheme (Table 5).

**Table 4:** Differences in classification between Evaluation 1 (established description) and 2 (adapted scheme)

	Established description (Evaluation 1)	Adapted scheme (Evaluation 2)
Differences *n* (% of total knees)	81 (47.4)	22 (12.9)
A versus B (% of total differences)	47 (58.0)	16 (72.7)
A versus C	8 (9.9)	1 (4.5)
A versus D	2 (2.5)	0 (0.0)
B versus C	19 (23.5)	2 (9.1)
B versus D	3 (3.7)	1 (4.5)
C versus D	2 (2.5)	2 (9.1)

**Table 5:** Inter-reader reliability for established description and the adapted scheme

Trochlear dysplasia classification	Established description (kappa, 95%CI)	Adapted scheme (kappa, 95%CI)
Dejour types A, B, C, and D	0.23 (0.11–0.34)	0.79 (0.75–0.83)
Low grade (A) versus high grade (B–D)	0.28 (0.13–0.43)	0.80 (0.75–0.85)
A and C versus B and D	0.20 (0.05–0.34)	0.76 (0.71–0.81)

Intra-reader reliability comparing the differences between Evaluation 2 and Evaluation 3 was almost perfect for both readers with kappa values between 0.88 and 0.95 (see Table 6).

**Table 6:** Intra-reader reliability for the adapted scheme

Trochlear dysplasia classification	Reader 1 (kappa, 95%CI)	Reader 2 (kappa, 95%CI)
Dejour types A, B, C, and D	0.90 (0.87–0.93)	0.92 (0.89–0.95)
Low grade (A) versus high grade (B–D)	0.88 (0.84–0.92)	0.95 (0.93–0.98)
A and C versus B and D	0.89 (0.86–0.93)	0.90 (0.87–0.93)

Inter-reader reliability analysis of spur and cliff size measurements yielded very good reliability (see Table 7). Both spur size and cliff size differed significantly (*p* < 0.001) between types B vs. D and C vs. D, respectively (Table 8). Both spurs and cliffs were significantly larger in Dejour type D.

**Table 7:** Inter-reader reliability for size of spur and cliff

Parameter	ICC (95% CI)	Sem
Spur size	0.917 (0.889, 0.938)	0.7 mm
Cliff size	0.923 (0.897, 0.943)	0.5 mm

*ICC*, intra-class correlation coefficient; *CI*, confidence interval; *SEM*, standard error of measurement

**Table 8:** Comparing inter-type spur sizes and cliff sizes based on measurements of reader 1: median (inter-quartile range) spur size between types

	Type			
Parameter	*B*	*C*	*D*	*P*-value
Spur size [mm]	3.0 (2.7, 3.5)	-	4.0 (3.0, 5.0)	< 0.001
Cliff size [mm]	-	3.5 (3.3, 3.7)	4.5 (4.0, 5.0)	< 0.001

*P*-values were calculated using Mann–Whitney *U*-tests

## Discussion

Radiologists’ inter-reader reliability for the Dejour classification was significantly higher in this study when using the adapted scheme. Intra-reader agreement was excellent for the adapted approach. This study is the first to use specified sagittal MR images for grading and also to introduce cut-off values for the size of a spur or cliff on MR images.

The original title of the paper describing the Dejour classification from 1998 was “Douleurs et instabilité rotulienne. Essai de classification” [6]. The article was published in the journal “la revue Médecine et Hygiène,” which was founded in Geneva, Switzerland, in 1943. Today, this journal is known as the “Revue Médicale Suisse.” The original description included conventional and cross-sectional criteria. The translation of the original description from French is as follows:

- Simple dysplasia: crossover sign but normal morphology of trochlear facets on CT
- Simple dysplasia with a supratrochlear spur: increased radiological prominence and an overall convex trochlea on CT
- Complex dysplasia: double contour ending below the crossing sign on plain radiography and hypotrophy of the medial side and convexity of the lateral side on axial CT scan
- Complex dysplasia with supratrochlear spur: increased radiological prominence and disappearance of the medial side and hypertrophy of the lateral side

However, the description of the criteria for analyzing the axial cross-sectional images and the graphical schemes have been modified repeatedly and therefore markedly changed over the years (current established description: see Table 1 [9]). In the novel adapted scheme, we have integrated criteria for the conventional X-ray images and simplified the criteria for sectional imaging including not only axial but also sagittal MR images.

The low inter-reader agreement with *k*-values between 0.11 and 0.43 in our first evaluation using the current established description [9, 22] is as expected from other recent studies [8, 10, 23], for instant the paper by Sharma et al. with *k*-values between 0.24 and 0.46 based on cross-sectional images [14]. The absolute number of inter-reader disagreement in our study was very high using the established description (in 47.4% of cases, so almost as reliable as tossing a coin) and significantly lower when using the adapted scheme (in 12.9% of cases). Both, for the established description and for the adapted scheme, most differences between the readers were seen between types A and B (58.0 versus 72.7%). Differences between types B and C were a major problem for the established description (23.5%, *n* = 19), but not for the adapted scheme (9.1%, *n* = 2). Trochlear dysplasia type A (= low grade) was the most common type when using the adapted scheme in our study cohort. This seems plausible since we searched the data base for all patients with a stated “trochlear dysplasia” in the knee MRI report and not for a subgroup of patients with known trochlear instability or incidence of patella dislocation.

In 2000, Pfirrmann et al. [24] found that the presence of trochlear dysplasia can be reliably diagnosed with MRI, but they did not further classify the severity of the dysplasia. Tscholl et al. [11] only found a fair agreement between trochlear dysplasia measured on radiographs and different levels on axial MR images, especially when the supratrochlear region of the distal femur is not analyzed on axial MRI. They concluded that an MRI evaluation limited to the cartilaginous trochlea alone tends to underestimate the severity of dysplasia. Therefore, we also included the distal femur in the analysis for signs of trochlear dysplasia and included not only axial, but also specified mid-sagittal MRI slices in our study.

Various other publications criticized that the reliability and reproducibility of Dejour’s classification is too low for its routine use in research and clinical practice [9, 22, 25]. Zimmerer et al. [26] underlined the need for a reliable and valid classification to grade trochlear dysplasia and recommended that the Dejour classification may be only grouped into low grade (type A) and high grade (types B to D). Another study by Fucentese et al. [1] modified Dejour’s classification combining patients with presence of a spur (types B and D) as high-grade and patients without spur presence (types A and C) as low-grade trochlear dysplasia. The authors stated that patients with a spur had better clinical outcomes after trochleoplasty regarding subjective knee pain and pain during sporting activities. Therefore, we tested in this study also the reliability of our adapted scheme regarding low-grade versus high-grade dysplasia and found excellent intra- and inter-observer reliabilities for both variants.

To date, there are no clear thresholds for the presence of a spur or cliff on MR images. A supratrochlear spur is defined as prominence of the trochlea above the anterior femoral cortex [9]. A clear spur indicates an elevation of the trochlear floor with loss of relative lateral trochlear height and increased susceptibility to patellar instability [9]. For the evaluation of spurs, we adapted the published measurement methods for sagittal radiographs to sagittal MR images [9, 27, 28]. To increase the specificity, we introduced a cutoff value of > 2.5 mm for spur since tiny bony irregularities (< 2 mm) are frequently found at the distal femur. Formation of osteoarthritis-related osteophytes at the distal femur is a minor differential diagnosis for spur presence since a spur is visible in the center of the trochlea, while the osteophytes usually occur at the trochlear edges. In orthopedic literature, Dejour types B and D with a spur larger than 5 mm are considered (together with other factors such as symptomatic patellar instability) as indications for proximal trochlear resection therapy [29]. A cliff is present with a missing or hypoplastic medial facet with a vertical transition from the lateral facet in the axial plane [9]. For the evaluation of a cliff, only a step-like transition from the lateral facet to anterior cortical bone of the femur > 3 mm was rated as a clear cliff in order to avoid overdiagnosis. We found a significant larger cliff and spur sizes for Dejour type D compared to types C and B. For the adapted scheme, the smaller spur size in types B compared to D might serve as an explanation, that the readers had some difficulties in differentiation between spur and no spur presence regarding types A and B, but no difficulties between all types and type D.

Recent studies tried to correlate and expand the Dejour classification with quantitative measurements (e.g., trochlear depth or lateral trochlear inclination [30, 31]) or 3D printing [32]. Some even introduced other classifications for the grading of trochlear dysplasia [14, 15, 33, 34]. But up to now no other classification system for trochlear dysplasia could be established in the orthopedic community, and referring physicians continue to ask for the Dejour classification in MRI reports. The proposed adapted scheme in this study might be used with both MR and CT images. However, since our data only included MR images, a further evaluation using CT images is recommended in the future.

In summary, the adapted scheme shows an almost perfect intra-reader reliability and a significantly higher inter-reader reliability than the established description and can therefore be a reliable tool for the daily diagnostic work of radiologists.

## Declarations

All procedures performed in studies involving human participants were in accordance with the ethical standards of the institutional and/or national research committee and with the 1964 Helsinki declaration and its later amendments or comparable ethical standards.
